# Non-Alcoholic Steatohepatitis (NASH) and Organokines: What Is Now and What Will Be in the Future

**DOI:** 10.3390/ijms23010498

**Published:** 2022-01-02

**Authors:** João Paulo Margiotti dos Santos, Mariana Canevari de Maio, Monike Alves Lemes, Lucas Fornari Laurindo, Jesselina Francisco dos Santos Haber, Marcelo Dib Bechara, Pedro Sidnei do Prado, Eduardo Costa Rauen, Fernando Costa, Barbara Cristina de Abreu Pereira, Uri Adrian Prync Flato, Ricardo de Alvares Goulart, Eduardo Federighi Baisi Chagas, Sandra Maria Barbalho

**Affiliations:** 1 Faculty of Medicine of Marilia (FAMEMA), School of Medicine, Avenida Monte Carmelo, 800, Marilia 17519-030, SP, Brazil; jpmargiotti95@gmail.com (J.P.M.d.S.); nana16032009@gmail.com (M.C.d.M.); 2 School of Medicine, University of Marília (UNIMAR), Avenida Hygino Muzzy Filho, 1001, Marilia 17525-902, SP, Brazil; monikealvesx3@gmail.com (M.A.L.); lucasffffor@gmail.com (L.F.L.); haber.jesselina@gmail.com (J.F.d.S.H.); dib.marcelo1@gmail.com (M.D.B.); uriflato@gmail.com (U.A.P.F.); 3 Interdisciplinary Center on Diabetes (CENID)-UNIMAR-Marília, Marilia 17525-902, SP, Brazil; efbchagas@gmail.com; 4 Hospital Israelita Albert Einstein Intensive Care Unit, Morumbi 05652-900l, SP, Brazil; pedro.prado@einstein.br; 5 Americas Serviços Medicos, Intensive Care Unit, Samaritano Hospital, Sao Paulo 01232-010, SP, Brazil; fernando.costa@samaritano.com.br (F.C.); barbara.abreu.pereira@gmail.com (B.C.d.A.P.); 6 Rauen Institute, Sao Paulo 05670-000, SP, Brazil; edurauen@gmail.com; 7 Postgraduate Program in Structural and Functional Interactions in Rehabilitation-UNIMAR-Marília–SP, Brazil, Avenida Hygino Muzzy Filho, 1001, Marilia 17525-902l, SP, Brazil; ricardogoulartmed@hotmail.com

**Keywords:** non-alcoholic fatty liver disease, dyslipidemias, oxidative stress, inflammation, organokines

## Abstract

Non-alcoholic steatohepatitis (NASH) is characterized by steatosis, lobular inflammation, and enlargement of the diameter of hepatocytes (ballooning hepatocytes), with or without fibrosis. It affects 20% of patients with non-alcoholic fatty liver disease (NAFLD). Due to liver dysfunction and the numerous metabolic changes that commonly accompany the condition (obesity, insulin resistance, type 2 diabetes, and metabolic syndrome), the secretion of organokines is modified, which may contribute to the pathogenesis or progression of the disease. In this sense, this study aimed to perform a review of the role of organokines in NASH. Thus, by combining descriptors such as NASH, organokines, oxidative stress, inflammation, insulin resistance, and dyslipidemia, a search was carried out in the EMBASE, MEDLINE-PubMed, and Cochrane databases of articles published in the last ten years. Insulin resistance, inflammation and mitochondrial dysfunction, fructose, and intestinal microbiota were factors identified as participating in the genesis and progression of NASH. Changes in the pattern of organokines secretion (adipokines, myokines, hepatokines, and osteokines) directly or indirectly contribute to aggravating the condition or compromise homeostasis. Thus, further studies involving skeletal muscle, adipose, bone, and liver tissue as endocrine organs are essential to better understand the modulation of organokines involved in the pathogenesis of NASH to advance in the treatment of this disease.

## 1. Introduction

Non-alcoholic fatty liver disease (NAFLD) can be defined as a macrovesicular steatosis (fat accumulation) in more than 5% of hepatocytes in the absence of a secondary cause such as alcohol and drug use or pre-existing liver disease. Non-alcoholic steatohepatitis (NASH) is a complication of NAFLD. In addition to steatosis, it is characterized by lobular inflammation and enlargement of the diameter of hepatocytes (ballooning hepatocytes), with or without fibrosis, and affects approximately 20% of patients with NAFLD. Such changes are noted on histological examination, and the presence of fibrosis reveals the worsening of the case. Its progression is related to cirrhosis, hepatocellular carcinoma, liver transplantation, and increased mortality from liver causes [1,2].

It should be noted that the metabolic definition of liver disease is primarily defined based on histopathological (pathological) findings or changes; however, recently, many hepatologists have suggested to rename NAFLD as MAFLD (metabolic associated fatty liver disease). The new term would be more faithful to the tight relationship between fatty liver and overfeeding, physical inactivity, and metabolic conditions (such as hypertension, DM2, dyslipidemia, and obesity) [3,4,5].

**Figure 1:**
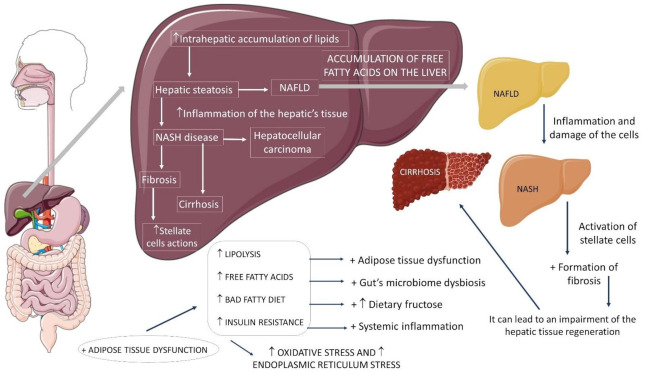
Non-alcoholic fatty liver disease and its progressions. ↑: increase, +: plus, NAFLD: non-alcoholic fatty liver disease, NASH: non-alcoholic steatohepatitis.

The understanding of the pathophysiology of NASH evolved substantially from the original hypothesis of two hits. The first is hepatic steatosis due to insulin resistance (IR). The second is oxidative stress, insufficient to describe the multiple molecular pathways and metabolic processes in NASH development. Thus, a multihit model was more recently proposed to explain this phenomenon, as it considers several injuries occurring in parallel in genetically predisposed individuals (polymorphisms in the PNPLA3 and TM6SF2 genes), such as nutritional factors, intestinal microbiota, IR, hormones secreted by adipose tissue, genetic factors, and epigenetics [2,6,7,8]. Figure 1 shows some aspects of NASH.

The prevalence of NASH is difficult to estimate due to the need for liver biopsy, the gold standard for diagnosis, and this practice is infrequent. According to indirect estimates, 3 to 6% of Americans present NASH, being more prevalent in individuals with previously established metabolic diseases, and of these, 20% progress to cirrhosis [2,9,10].

Considering that NAFLD (the precursor of NASH) affects up to 1 billion people worldwide and its prevalence has accompanied the growth of cases of obesity and Metabolic Syndrome (MS), it is projected that the set of both conditions will be responsible for the main causes of liver transplantation [6,10].

Concerning etiology, the primary agents associated with NAFLD and NASH are the presence of insulin resistance (IR), type 2 diabetes (DM2), obesity, and MS. Other endocrine diseases, such as hypothyroidism and polycystic ovary syndrome, can be associated with this condition since they share the fundamental pathophysiological mechanism of NAFLD: IR, obstructive sleep apnea, hypertension, dyslipidemia, changes in the intestinal microbiota, genetic predisposition, menopause, sedentary lifestyle, and excessive consumption of fructose, saturated fatty acids and carbohydrates [2,11,12].

Due to liver dysfunction, the secretion of hepatokines also changes. Moreover, the associated metabolic changes alter the secretion of other organokines, which act in the modulation of NASH, either in its pathogenesis or in the progression of the disease [13,14]. For these reasons, this study aimed to review the role of organokines in NASH.

## 2. Insulin Resistance, the Role of Fructose, Gut Microbiota and Organokines

### 2.1. Insulin Resistance, Inflammation, and Mitochondrial Dysfunction

IR has a crucial role in the pathophysiology of NAFLD/NASH. Usually, insulin has a regulatory function of hepatic metabolic processes. It acts in other peripheral cells, favoring glucose uptake, storage pathways (glucogenesis), protein, and fatty acid synthesis. On the other hand, it blocks catabolic processes such as lipolysis, proteolysis, glycogenolysis, and gluconeogenesis. Therefore, resistance to its action generates several harms to the metabolism [15,16,17].

With overnutrition (marked by high consumption of carbohydrates, lipids, and fast foods) and a sedentary lifestyle, excessive energy intake is stored in the adipose tissue. By absorbing and storing excess glucose and free fatty acids (FFA), adipose tissue, as a compensatory mechanism, neutralizes the potentially toxic effects of these circulating nutrients, which include adipocyte hypertrophy and hyperplasia. In line with overnutrition, IR increases the flow of fatty acids to the liver by boosting hepatic lipogenesis and lipolysis, and inhibiting FFA esterification, generating an imbalance between synthesis/input versus oxidation/exportation of hepatocellular fat [6,18,19,20,21,22].

Thus, such imbalance is responsible for the excessive intrahepatic accumulation of fatty acids. This accumulation is lipotoxic, as the cells, already incapable of sequestering more reactive lipid molecules, have their mitochondrial beta-oxidation enzymatic system overloaded and suffer mitochondrial damage, endoplasmic reticulum (ER) stress, and autophagy. The results of obesity associated with NASH lead to hypertrophy of adipocytes, cell degeneration and death [2,6,23,24].

The death of hepatocytes is related to the release of damage-associated molecular patterns (DAMPs) to neighboring cells, which, through the inflammasome, are converted into pro-inflammatory cytokines (IL-1β and IL-18). Once secreted, IL-1β attracts neutrophils and activates both neutrophils and hepatic stellate cells (HSCs) and, in turn, IL-18 secretion attracts and activates macrophages. Such responses occur because DAMPs stimulate innate immunity through binding to pattern recognition receptors (PRRs) that activate NFκβ (nuclear factor κβ), initiating the pro-inflammatory action in NASH. Its release results in a cascade of pro-inflammatory chemokines and cytokines, such as TNF-α and IL-6, culminating in ROS formation. The combination of this scenario with liver sinusoidal endothelial cells (LSECs) have a direct action on fibrosis, in addition to providing an environment of chronic regeneration, favoring chromosomal aberrations and, therefore, the development of hepatocellular carcinoma [6,25,26,27]. 

The increased concentration of ROS can induce cell death in hepatocytes by activating specific pathways and inducing lipid peroxidation at the expense of β-oxidation, resulting in the synthesis of reactive lipids. These reactive molecules can further amplify liver damage, promote the release of more ROS outside the hepatocytes, and contribute to the activation of HSCs and extracellular matrix deposition [24,28].

The peroxidation process generates malondialdehyde, which is also responsible for perisinusoidal and periportal fibrosis, by activating NFκβ and regulating the expression of pro-inflammatory cytokines such as TNF-α and IL-8. These inflammatory biomarkers activate HSCs, contributing to fibrosis. The peroxidation of membrane phospholipids alters their permeability and promotes ballooning hepatocytes. This change may reflect cytoskeletal damage with the inability to complete programmed cell death. This change causes the accumulation of hydrogen peroxide in hepatic peroxisomes, which, together with ferrous iron, produce free hydroxyl (OH) radicals, which are very reactive with membrane phospholipids [24,29,30].

Lipotoxicity is associated with the transition of mitochondrial membrane pores that causes interruption of cellular respiration, generation of oxidative stress, and cytochrome C extravasation from the matrix to the cytosol, activating the apoptosome in apoptosis. Mitochondrial DNA is hit by the excess of free radicals, causing reduced oxidative phosphorylation and depleting ATP stores. The inhibition of electron flows in the phosphorylation chain generates more ROS, further damaging the mitDNA, creating a vicious circle. Therefore, apoptosis is aborted when ATP is depleted, and necrosis or necroptosis occurs—a mechanism involving mitochondrial damage, oxidative stress, and the activation of the c-Jun N-terminal kinase (JNK) [24,25,31,32].

Therefore, various pathological stimuli, including hepatocyte death, molecules secreted by adipose tissue, and intestinal pathogens, can promote inflammation and fibrogenesis by activating resident macrophages (Kupffer cells). Both Kupffer cells and obesity play a critical role in the pathophysiology of NASH. They promote the activation of the phenotype of pro-inflammatory M1 macrophages to the detriment of non-inflammatory M2 macrophages. Activated M1 macrophages produce a variety of cytokines that recruit pro-inflammatory cells, amplifying inflammation. This complex series of events ultimately culminates in the activation of HSCs, followed by excessive synthesis and extracellular matrix deposition. Thus, the loss of function of adipocytes added to adipose inflammation (presence of IL-1, IL-6, and TNF-α) contributes to the development of adipose resistance to insulin [10,23,28,29].

### 2.2. The Role of Fructose

The role of fructose in NASH is also noteworthy, as it stimulates the accumulation of hepatic fat and is related to the MS risk factors such as high blood pressure, elevated serum triglycerides, and IR. It is noteworthy that the origin of fructose influences this process. The consumption of sweetened fructose drinks and the consumption of fruits show different results, as the latter have a lower fructose content, in addition to containing antioxidants, which combat the effect of this monosaccharide [33,34,35].

**Figure 2:**
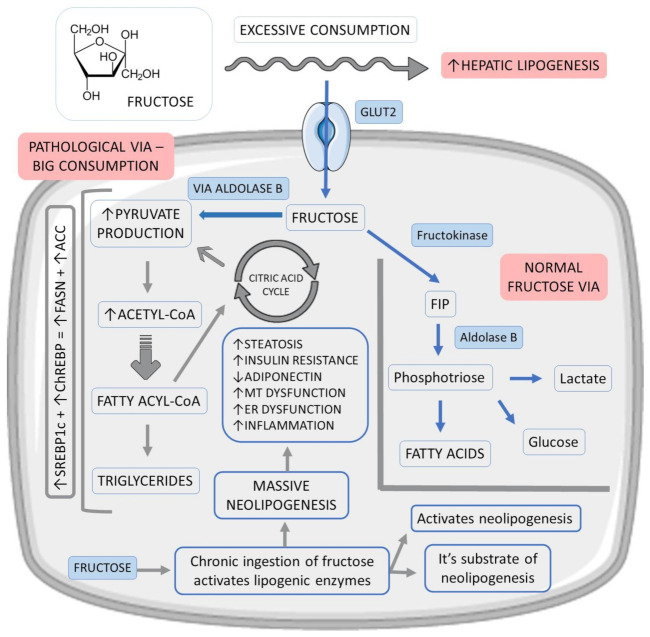
Fructose vias in hepatocytes and their relations with fat liver accumulation. ↑: increase, ↓:decrease, +: plus, ACC: acetyl-CoA carboxylase, ChREBP: carbohydrate-responsive element-binding protein, ER: endoplasmic reticulum, FASN: fatty acid synthase, FIP: fructose-1-phosphate, GLUT2: glucose transporter 2, MT: mitochondrial and SREBP1c: sterol response element-binding protein 1c.

Fructose enters the hepatocyte rapidly via glucose transporter 2 (GLUT2) and, at the cellular level, is preferentially converted to fructose-1-phosphate (F1P) by fructokinase. F1P then undergoes the action of aldolase B, producing phosphotriosis, which can be converted into glucose, lactate, and fatty acids. After an acute fructose load, the lipogenic pathway, often underactive, becomes very active as the flow of phosphotriosis increases [29,36]. Figure 2 shows the metabolism of fructose.

In addition, dysregulation in the hepatic entry and metabolism of fructose culminates in excess production of acetyl-CoA (glycolytic pathway), above the liver’s oxidative capacity, promoting neolipogenesis through the activation of factors such as sterol response element-binding protein 1c (SREBP1c) and carbohydrate-responsive element-binding protein (ChREBP). As a product of the activation of these factors, there is an increase in the expression of fatty acid synthase (FASN) and acetyl-CoA carboxylase (ACC), which regulate lipid synthesis. Saturation of the glycolytic pathway also promotes an accumulation of glycolysis intermediates, which can be converted into glycerol-3-phosphate, used in triglyceride synthesis (TG) [36,37].

Therefore, fructose stands out as the most potent lipogenic carbohydrate in the development of hepatic steatosis since it is both a substrate and an activator of neolipogenesis. Chronic ingestion of this carbohydrate increases this process by stimulating the expression of lipogenic enzymes. The stimulation of neolipogenesis and the accumulation of lipids in the liver can increase the hepatic IR. Furthermore, fructose is also known to inhibit adiponectin production and release, contributing to increased IR and, therefore, potentiating hepatic steatosis. Such events can promote oxidative stress due to mitochondrial dysfunction and endoplasmic reticulum stress, which are factors that stimulate inflammation and progression from simple steatosis to NASH [36,38,39].

Upon entering the liver, fructose is rapidly phosphorylated by fructokinase C without negative feedback control. This process reduces the production of adenosine triphosphate (ATP) and leads to its consumption because while phosphorylation by fructokinase is fast, the cleavage reaction by aldolase B is relatively slow [36]. Thus, excess fructose, that is, consumption greater than that recommended by the World Health Organization (WHO) which is 5% in relation to the total recommended daily calories, can cause liver phosphate deficiency, with the accumulation of adenosine monophosphate (AMP) and consequent increase in uric acid synthesis, with fructose being the only common carbohydrate that produces uric acid in its metabolism [36,40]. The reduction of intracellular phosphate activates the enzyme AMP deaminase, which converts AMP into inosine monophosphate (IMP), resulting in a nucleotide renewal that stimulates the formation of uric acid, contributing to the formation of ROS [33,38,39].

In this sense, it is significant to observe that even though glucose and fructose are isomers and therefore have the same molecular formula (C6H12O6), the beginning of the metabolism of these molecules differs. The major difference is the involvement of phosphofructose kinase (a regulatory enzyme) in glucose metabolism but not in fructose metabolism. This initial difference leads to decreased intracellular phosphate and ATP levels, and uric acid generation is sufficient to transiently block protein synthesis, induce ROS, and mitochondrial dysfunction, resulting in an MS phenotype. From this stage onwards, the metabolism of these carbohydrates is similar [33,41,42].

The accumulation of uric acid can impair fatty acid oxidation, reduce hepatic ATP levels, and cause the temporary blockage of protein synthesis, inducing oxidative stress and mitochondrial dysfunction. In addition, uric acid also has direct pro-inflammatory effects by activating NFκβ and stimulating the release of inflammatory cytokines. Finally, fructose stimulates the rupture of tight intercellular junctions, contributing to greater intestinal permeability, which allows the entry of endotoxins through the portal vein, worsening the condition of NASH [33,38,43].

In addition, mitochondria contain enzymes known to be sensitive to oxidative stress (aconitase-2 and enoyl CoA hydratase). Together, fructose and uric acid reduce the action of aconitase-2, an enzyme associated with the Krebs cycle, which causes the accumulation of citrate. This excess of citrate is directed to the cytoplasm and activates lipogenesis by stimulating the enzyme ATP citrate lyase. It is also relevant to note that repeated exposure to sugar stimulates glucose transport via GLUT-5 and increases the levels of fructokinase in the liver, resulting in higher absorption of fructose [33,34,44,45].

Excessive and chronic consumption of fructose due to a high glycemic index diet, leads to increased demand for the endoplasmic reticulum (ER) due to the stimulation of lipid metabolism. Therefore, endoplasmic reticulum membrane proteins may be fructolyzed or accumulate lipids contributing to ER stress, generating inflammation, oxidative stress, and apoptosis. ER stress also indirectly stimulates TG accumulation in the liver, inducing hepatic and adipose IR and activating transcription factors related to inflammation and cell death. Fructose promotes the synthesis of saturated fatty acids, which can activate toll-like receptor 4 (TLR4) in the liver. This activation induces oxidative stress resulting from the production of inflammatory cytokines. Furthermore, ER stress and inflammation can lead to the production of DAMPS that signal and contribute to changes throughout metabolism. Excess fructose can change the profile of organokines, with increased production of fetuin A, fibroblast growth factor 21 (FGF-21), leucocyte cell-derived chemotaxin 2 (LECT2) and angiopoietin-like protein (ANGPTL), involved in changes in energy homeostasis, in IR and that can contribute to peripheral organ damage [6,36,46].

### 2.3. Intestinal Microbiota

The liver receives most of the intestinal blood flow through the portal system. Therefore, it is the first line of defense for intestinal-derived toxins and is exposed to many pathogen-associated molecular patterns (PAMPs); hence, the gut microbiota role in the pathophysiology of NASH is important. Excessive ingestion of foods high in fat or sugar is associated with a significant depletion of bacterial species diversity, with a reduction in the total density of the gut microbiota. In the NASH scenario, the major changes in some groups of bacteria are impressive. This pathology can produce severe depletion of bacterial species diversity, accompanied by a marked reduction in the total density of the microbiota. As a result, beneficial commensal bacteria may have been lost, and pathological microbes are overrepresented. Some examples of beneficial microorganisms are *Bifidobacterium, Lactobacillus, Faecalibacterium, Roseburia, Ruminococcus, Bacteroides* sp., which have anti-inflammatory effects and favorable action on metabolic patterns. Bacteria that can represent damage to the intestinal microbiota are *Clostridium, Enterobacter,* and *Enterococcus* sp. Patients with NASH reduce the amount of bacterioides compared to healthy individuals [47]. Dysbiosis favors the passage of bacteria and bacterial products (endotoxins), such as lipopolysaccharides (which may be related to the development of fibrosis by activation of hepatic stellate cells) to the portal circulation thus contributing to the activation of hepatic stellate cells to the progression of NAFLD to NASH. Diet-induced obesity alters the expression and distribution of tight junctions between intestinal cells, which is directly associated with increased intestinal permeability. Furthermore, patients with NASH compared to healthy or obese patients have an excess of ethanol-producing bacteria, specifically *Escherichia*, indicating that ethanol-producing microorganisms (*Bacteroides, Bifidobacterium*, and *Clostridium* sp.) can act as a risk factor for NASH progression. Therefore, it is notorious that there are pathologies that alter the intestinal microbiota, but its composition is also affected by the diet [24,28,38,47,48,49,50].

In this mechanism, a substance that deserves attention is fructose. It stimulates changes in the composition of the small intestine microbiota, reducing the expression of cell junction proteins and interfering with cell adhesion. Such processes allow the entry of endotoxins through the portal vein, triggering upregulation of lipogenic genes, as well as pro-inflammatory genes. This higher permeability allows bacterial translocation from the intestine to the bloodstream, providing pathogenic signals to various organs, including the liver, an event that can stimulate the innate immune response and further increase the pro-inflammatory response [24,38,51,52]. 

Scientific evidence indicates that changes in lifestyle—with adherence to diet and exercise—and reduction in body weight improve NAFLD. Increased dietary fiber intake, probiotics and prebiotics, and caloric restriction are examples of recommendations. Dietary fiber is the main non-digestible element in most diets, influencing the modulation of digestion as a substrate for microbial fermentation. Furthermore, the degradation of fibers that act as prebiotics provides short-chain fatty acids, which act as protectors of the structure and function of the intestinal barrier. A high-fiber diet increases the amount of *Bifidobacterium* and decreases *Firmicutes/Bacteroidetes* in humans, in addition to reducing the frequency of feeding by increasing satiety, which contributes to caloric restriction as a factor in improving NASH. Some studies show that a higher intake of insoluble fiber (≥7.5 g/day) can improve three different liver fibrosis scores (hepatic fibrosis index, fatty liver index, and liver fat index NAFLD) [24,47,53].

### 2.4. The Role of Diacylglycerols

Diacylglycerols (DAGs) also play an essential role in the pathophysiology of NASH, causing hepatic IR and the progression of liver steatosis. DAGs are the penultimate intermediates for triglyceride production and have been investigated to mediate IR among hepatocytes by activating protein kinase C epsilon type (PKCƐ). PKCƐ phosphorylates a threonine residue on insulin receptors of hepatocytes, impairing insulin signaling in these cells and leading to IR-derived lipid deposition stimuli. Moreover, in individuals with NAFLD and NASH, DAGs can be expressed in the liver under higher concentrations, augmenting triglycerides production extensively. Increased hepatic IR and liver lipid production are assessed as DAGs-mediated contributors of liver fatty accumulation and steatohepatitis [54,55]. In a trial, Loomba et al. [56] evaluated if the antisense inhibition of diacylglycerol O-acyltransferase 2 (DGAT2) could effectively reduce liver fatting in individuals with diabetes and NAFLD and showed that the inhibition reduced liver fat content without causing hyperlipidemia. 

## 3. Organokines

The endocrine function of the liver, adipose, and hepatic tissues is of great value in NASH development. These tissues can produce biomarker peptides named organokines (hepatokines, adipokines, and myokines, respectively) that crosstalk through autocrine, endocrine, and paracrine pathways. The combined action of organokines is related to health or to the genesis of several diseases. With the increase in adipose tissue, there is an increase in the secretion of pro-inflammatory organokines, contributing to metabolic disorders such as IR, DM2, and MS [13,48].

The proximate relationship between skeletal muscle and bone tissue goes beyond anatomy: these tissues are also physiologically connected by the endocrine system through organokines, which completes this biochemical crosstalk. As discussed above, when there is a chronic excess of energy intake, there is an increase in adiposity and lipid deposition both in the bone marrow and in nearby myocytes, causing the release of FFA, which are lipotoxic to muscle and bone cells present there. This process triggers a vicious cycle, with low-grade systemic inflammation (LGI) and impairing metabolism. The load applied to skeletal muscle in resistance exercise is transferred to bone, which in addition to initiating muscle protein production, also signals a high energy requirement to facilitate bone formation. Some systemic mediators, including leptin, can initiate muscle hypertrophy and bone formation, proving that such tissues receive endocrine signals, and bone and muscle are now a bidirectional pathway for biochemical signals. Such signals are commanded by myokines, osteokines and adipokines, exerting autocrine, paracrine, and endocrine effects, thus regulating muscle and bone metabolism [14,57].

Since the liver is a producer of essential hormones or hormone precursors, such as insulin-like growth factor I (IGF-1), angiotensinogen, thrombopoietin, and hepcidin, the lipid imbalance resulting from the damage and death of hepatocytes is also responsible for the dysregulation of production of organokines. The expression of the insulin-sensitizing anti-inflammatory hormone adiponectin is decreased in NASH. Although leptin is essential to regulate appetite and increase energy expenditure, its levels are increased in obesity. Leptin promotes fibrogenesis in stellate cells, stimulating the production of fibrogenic genes and inflammation in T cells [16,58].

Several myokines, such as irisin, IL-6, IGF-1, brain-derived neurotrophic factor (BDNF), and myostatin, exert effects on bone metabolism, both anabolic and catabolic. In contrast, osteokines such as osteocalcin and sclerostin induce muscle anabolism and catabolism, respectively. Adipokines such as resistin, leptin, adiponectin, and TNF-α can also interfere with bone and muscle metabolism. Furthermore, lipolytic myokines such as irisin and IL-6 released after exercise stimulate thermogenesis and darkening adipocytes [57,59].

### 3.1. Adipokines

In addition to its role of storing energy, contributing to thermoregulation, and exerting mechanical protection, the adipose tissue also plays a role as an endocrine organ, secreting bioactive peptides called adipokines [60]. These substances regulate lipid metabolism and influence insulin sensitivity, appetite, fibrogenesis, and fat accumulation in the liver. Their action is directly related to adipocyte hypertrophy, primarily in obesity, and is associated with a mild inflammatory condition [59]. Besides being involved in the pathogenesis of some metabolic diseases, they modulate bone turnover, bone mineral density, as well as skeletal muscle catabolism in aging, such as sarcopenia. The accumulation of visceral fat is related to increased levels of adipokines [57].

#### 3.1.1. Adiponectin

The greater the expression of adiponectin, the smaller the adipose tissue mass. Such adipokine has anti-inflammatory actions, increases fatty acid oxidation and glucose uptake in skeletal muscle, and inhibits hepatic gluconeogenesis [57]. Adiponectin is produced only by adipose tissue, stimulates the secretion of anti-inflammatory cytokines (IL-10), blocks the activation of NFκβ, and inhibits the release of TNF-α and IL-6. Weight loss induces its synthesis [61,62,63,64,65]. It is an antisteatotic, antifibrotic and anti-inflammatory substance, being expressed by both white and brown adipocytes. Its reduction is directly related to IR and glucose intolerance. Its levels can be lowered with the increase of pro-inflammatory cytokines. In addition to contributing to inflammation, such adipokine induces thermogenesis and fatty acid oxidation in skeletal muscle and liver [64,66]. Moreover, adiponectin may contribute to keeping HSCs in their quiescent state [65]. Thus, adiponectin can be considered a protective adipokine. Its levels are inversely related to RI and lower in obese individuals and patients with RI, for example, those with DM2, NASH, and hypertension [63].

#### 3.1.2. Leptin

In chronic low-grade inflammation and obesity, leptin stimulates production of IL-6 and TNF-α and reduces adiponectin. Excess leptin results in resistance to adiponectin, which limits the oxidation of muscle FA and reduces lipolysis in adipose tissue, effects that are neutralized by exercise [57]. Leptin is related to food intake and energy expenditure and its effects on NAFLD may be related to IR or failure in the antisteatotic effect. This adipokine has its secretion increased by IL-1 and TNF-α [63,64]. Leptin increases glucose and FFA absorption in skeletal muscle, in addition to reducing the amount of intrahepatic lipids due to fatty acid oxidation. The increased levels of leptin contribute to the action of pro-inflammatory cytokines. Furthermore, leptin stimulates the division of stellate liver cells and the production of both pro-fibrinogenic and pro-inflammatory factors. High levels of leptin have been shown to be associated with the progression of NAFLD [64,67]. Finally, this adipokine can exert a pro-inflammatory activity also due to the compromised relaxation of the nitric oxide (NO)-related vessel, via increased oxidative stress and by the increased expression of endothelin, potentiating the effect of angiotensin II, which, in turn, increases the synthesis of leptin further inducing the release of pro-inflammatory biomarkers (such as IL-6, TNF-α, and MCP-1 receptor), increasing the expression of adhesion molecules (vascular adhesion molecule 1 (VCAM-1), intercellular adhesion molecules (ICAM–1), and E-selectin) [63,67].

#### 3.1.3. Omentin

Omentin improves insulin sensitivity and consequently glucose absorption. Its main circulating form is omentin-1 (intelectin-1, an adipokine peptide with 313 amino acids). Patients with impaired glucose tolerance and newly diagnosed with DM2 have their levels reduced, contributing to arterial calcification. Its levels are inversely related to obesity, hyperglycemia, chemerin levels, inflammation, and insulin resistance, factors that favor the progression of NASH [68,69]. It can work as an anti-atherosclerotic and protect against liver diseases [64].

#### 3.1.4. Resistin

Resistin is related to IR in obesity. It has a pro-inflammatory action, stimulating the release of TNF-α and IL-12 [9,58,65]. It also induces angiogenesis and smooth muscle cell proliferation [9]. Resistin reduces the number of mitochondria and increases fat accumulation. Its expression was shown to be higher in patients with NASH compared to simple steatosis and control subjects [65,70]. It is produced by adipose tissue macrophages and monocytes, induces IR, angiogenesis, and smooth muscle cell proliferation [71].

#### 3.1.5. Vaspin

Vaspin is produced by visceral adipose tissue and is considered a member of the serine protease inhibitor family. It also has a relationship with the inhibition of kallikrein 7, a protease capable of degrading insulin. For these reasons, it can improve glucose intolerance, insulin sensitivity and can reduce the synthesis of pro-inflammatory cytokines, protecting vascular tissues from apoptosis. High serum levels are related to obesity, insulin sensitivity, physical conditioning, and low levels of leptin [60,64].

#### 3.1.6. Visfatin

Visfatin is produced mainly by adipocytes and macrophages in adipose tissue and to a lesser extent by hepatocytes and neutrophils. It is involved in the transformation of preadipocytes into adipocytes, in addition to stimulating the production and storage of triacylglycerols in adipose tissue [68]. It has immunomodulatory and vasodilatory properties, as it increases the synthesis of NO. On the other hand, it shows pro-inflammatory actions, promoting dysfunction of pancreatic β cells, activation of leukocytes and production of pro-inflammatory cytokines in adipose tissue, resulting in the release of TNF-α, VCAM-1, IL-6, and IL1β, factors that impair insulin signaling [64,65,68]. Furthermore, visfatin stimulates endothelial proliferation and the growth of smooth muscle cells. Its levels are increased in obesity, diabetes, MS, or cardiovascular disease (CVD) [68].

### 3.2. Myokines

Skeletal muscle is responsible for releasing myokines that can influence metabolic activities. This release is directly related to regular physical exercise [72]. These organokines show how the muscle has a critical secretory function, releasing peptides, cytokines, and growth factors. The type of myokine released is related to physical activity or its absence, in addition to the type of muscle fiber and the exercise performed [64].

#### 3.2.1. Irisin

Irisin is related to the regulation of energy homeostasis and metabolism, and in the interactions between skeletal muscle and other tissues. It can induce the differentiation of white adipose tissue (WAT) into brown adipose tissue (BAT) [63]. Such differentiation to brown adipose tissue suppresses adipogenesis and cholesterol synthesis, optimizing lipid oxidation and, consequently, lipid homeostasis as a whole. It is usually secreted after physical exercise and seems to increase insulin sensitivity, as it stimulates the mobilization of the glucose transporter in insulin-dependent tissues, improving MS and CVD, in addition to having an anabolic effect on bones, increasing the production of osteoblasts [57,64,73].

Polyzos et al. [74] detected higher levels of this myokine in lean patients than in obese patients and patients with NASH or NAFLD. Paradoxically, this study identified that, depending on the evolution of portal inflammation, an increase in irisin may occur, probably as a compensatory mechanism to limit inflammation.

#### 3.2.2. Mionectin

Mionectin is released in resistance exercises, increases lipid uptake by adipose tissue and the liver, reducing plasma FFA concentration. Physical inactivity reduces its levels, increasing the level of fatty acids in the blood, which causes their accumulation in other tissues and generates insulin resistance [64]. Furthermore, it affects the absorption of FFA by cells and integrates metabolic processes in various tissues [72].

#### 3.2.3. Myostatin

Myostatin facilitates muscle loss as it inhibits factors that promote tissue growth, in addition to stimulating tissue degradation, promoting skeletal muscle atrophy. It is essential to emphasize that reducing muscle mass increases metabolic disorders, increasing the RI and hepatic fat deposition. The skeletal muscle acts as the main source of glucose-dependent glucose uptake, and when muscle mass is lost, this deposition is compensated in other organs, such as the liver. With the practice of exercises, its levels are reduced; therefore, physical activity plays a relevant role as a therapeutic target. Its expression is higher in obese individuals and is closely linked to insulin resistance, promoting down-regulation of GLUT 4 expression. It is elevated in pro-inflammatory environments and inhibits the release of antioxidant and anti-inflammatory compounds. Its reduction directly causes a drop in RI, less accumulation of fat, generating muscle hypertrophy with positive regulation of the oxidative metabolism of skeletal muscles, lipolysis in the WAT and darkening of adipocytes, redistributing the FFAs used in lipogenesis for use as energy fuel, factors that prevent the progression of NASH [64,75,76].

**Figure 3:**
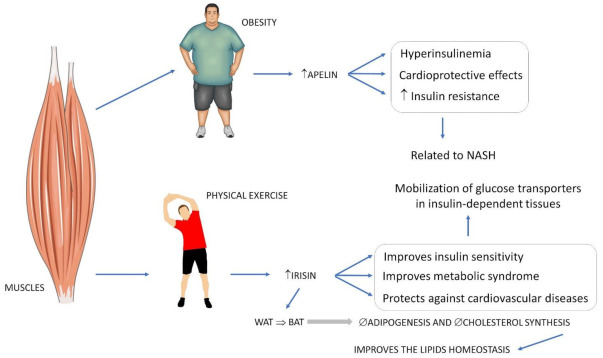
Some myokines and their relations to key factors involved in NASH. ↑: increase, ↓: decrease, ∅: restriction, WAT: white adipose tissue, BAT: brown adipose tissue.

Figure 3 shows the relationship between exercise, the release of myokines and NASH.

### 3.3. Hepatokines

Hepatokines play an essential role in modulating metabolic progress and pathological conditions, primarily as liver-derived pro-inflammatory factors [77]. Fetuin-A, fibroblast growth factor-21 (FGF-21), selenoprotein P, sexual hormone binding globulin (SHBG), angiopoietin like 4 (ANGPTL4) and leukocyte cell-derived chemotaxin (LECT2) are the most studied to date. The liver can affect lipid and glucose metabolism by releasing such organokines into the blood, and NAFLD appears to be associated with their altered production. Furthermore, hepatokines can be considered biomarkers of ectopic fat accumulation in the liver and markers of disease progression. Some may be the target for prevention and treatment of diseases associated with insulin resistance, including DHGNA [58,78].

#### 3.3.1. Angiopoietin-Like 4 (ANGPTL 4)

ANGPTL 4 is released by the liver during exercise. As it inhibits pancreatic lipases, it promotes less fat absorption. In white adipose tissue, it stimulates lipolysis and reduces the growth of this tissue. Its increase is directly linked to IR and MS [16,64], and it has a role in redistributing FFAs in skeletal muscle rather than storing in adipose tissue. In obese individuals, ANGPTL-4 is reduced. Thus, inhibition of lipoprotein lipase (LPL) is affected, causing lipid accumulation and the possible evolution of NASH [75,79].

#### 3.3.2. Leukocyte Cell-Derived Chemotaxin 2 (LECT2)

LECT 2 is a neutrophil chemotactic organokine, directly related to metabolic stress, as it impairs insulin signal transduction and increases inflammatory cytokines and adhesion molecules. Its absence increases insulin sensitivity [16,64].

In addition, it can detect hepatic steatosis and positively regulate itself through the activation of lipopolysaccharide (LPS) signaling in macrophages, preceding weight gain. Its expression is directly proportional to overnutrition and, in turn, to the body mass index (BMI) and liver inflammation, together with the development of NASH [80].

#### 3.3.3. Sexual Hormone-Binding Globulin (SHBG)

SHBG is a protein released primarily in the liver and transports sex steroids to its target tissues. Its levels are lower in DM2, obese patients and individuals with hepatic steatosis when compared with individuals with no liver pathology. The reduced expression of SHBG in NAFLD may occur secondary to inflammation, as an increase in TNF-α in response to JNK and NF-kB activation reduced SHBG production. With respect to lipid metabolism, SHBG overexpression protected against NAFLD development by inhibiting hepatic lipogenesis through the downregulation of key lipogenic enzymes. Modifications in lifestyle, such as therapy for obesity and weight reduction, caused an increase in circulating SHBG. Furthermore, adiponectin increases the production of SHBG by activating AMPK and reducing the activity of enzymes involved in liver lipogenesis. In other words, when the liver SHBG production is reduced as a result of the increase of pro-inflammatory cytokines, the hepatic lipogenesis will be favored. This fat accumulation in the liver will further reduce hepatic SHBG production, which, in turn, will increase hepatic lipogenesis, thus initiating a vicious cycle that exacerbates the development of NAFLD [16,64,81].

SHBG production is disrupted when liver damage occurs, as is the case with other sex steroid hormones. Thus, its levels are reduced in cases of steatosis, obesity, and DM2. In polycystic ovary syndrome, the high levels of androgens are responsible for the decrease in their production, which favors the onset or progression of liver diseases, including NAFL and NASH, and metabolic diseases [82].

**Figure 4:**
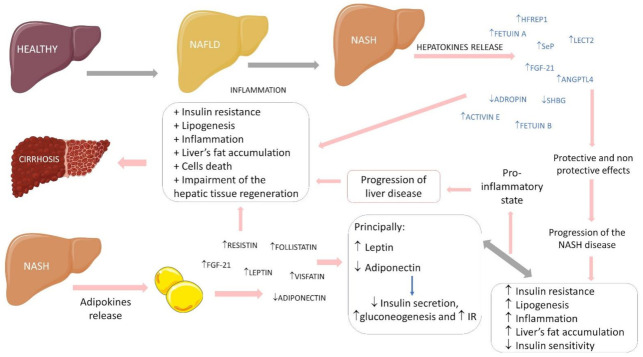
Relations between hepatokines and adipokines in the progression of fatty liver diseases. ↑: increase ↓: decrease, +: plus, HFREP1: hepatocyte-derived fibrinogen-related protein 1, SeP: selenoprotein-P, LECT2: leukocyte cell-derived chemotaxin 2, FGF-21: fibroblast growth factor 21, ANGPTL4: angiopoietin-like 4, SHBG: sexual hormone binding globulin, IR: insulin resistance, NASH: non-alcoholic fatty liver disease and NAFLD: non-alcoholic steatohepatitis.

Figure 4 shows some associations observed between hepatokines and adipokines in the progression of fatty liver diseases.

### 3.4. Osteokines

The release of osteokines either by direct stimulation of osteocytes and osteoblasts or by the release of muscle matrix factors generates a secondary response in the bone and other tissues, with autocrine, paracrine, and endocrine actions. Such osteokines have systemic effects, and some of them are osteocalcin (OCN) and bone morphogenetic protein (BMP). Osteocalcin, sclerostin, and BMP are released by active osteoblasts and can be released into the matrix during bone resorption. Moreover, they play relevant effects on metabolism [60,75].

#### 3.4.1. Bone Morphogenetic Protein (BMP)

BMP is a member of the TGF-β family and regulates several processes, such as organogenesis, patterns of embryonic development, and white and brown adipocyte production and energy expenditure. The expression of its subtypes in adipose tissue and its blood concentrations are linked to obesity and fat distribution. BMP-7 is involved in appetite regulation, decreasing food intake the higher its level. Furthermore, the BMP-4 subtype, secreted by adipocytes, stimulates adipogenesis and directs preadipocytes to a brown adipocyte phenotype. In obesity, preadipocytes are resistant to such BMP, which may contribute to obesity-related diseases, like NASH [60].

Furthermore, BMP-7 stimulates insulin secretion by the pancreas. Therefore, due to its importance in regulating food intake, energy expenditure and adipose tissue production, this class is essential for the improvement of obesity and its related comorbidities [57,83].

#### 3.4.2. Osteocalcin (OCN)

OCN is synthesized and released mainly by osteoblasts and then activated by osteoclasts in bone resorption [75]. Its non-carboxylated and subcarboxylated (ucOCN) forms increase insulin sensitivity and secretion by direct stimuli in the pancreas. ucOCN increases after exercise and thus stimulates glucose uptake and increases insulin sensitivity, and is related to muscle hypertrophy and strength. Non-carboxylated osteocalcin improves insulin sensitivity, glucose uptake, and IL-6 sensitivity in skeletal muscle in rats but has obscure effects in humans [57]. OCN levels are inversely related to BMI, IR, inflammatory markers, and body fat mass. It promotes the survival and functioning of pancreatic β cells, increasing insulin secretion, which itself induces the release of OCN, thus forming a direct endocrine relationship between bone and pancreas. Furthermore, it has beneficial effects as it stimulates the production of adiponectin and IL-10. On the other hand, it reduces TNF-α levels in adipocytes and positively regulates thermogenesis in adipose tissue and mitochondrial production in skeletal muscle. Besides that, it increases the glucose and FFAs uptake by muscles [75,84]. 

**Figure 5:**
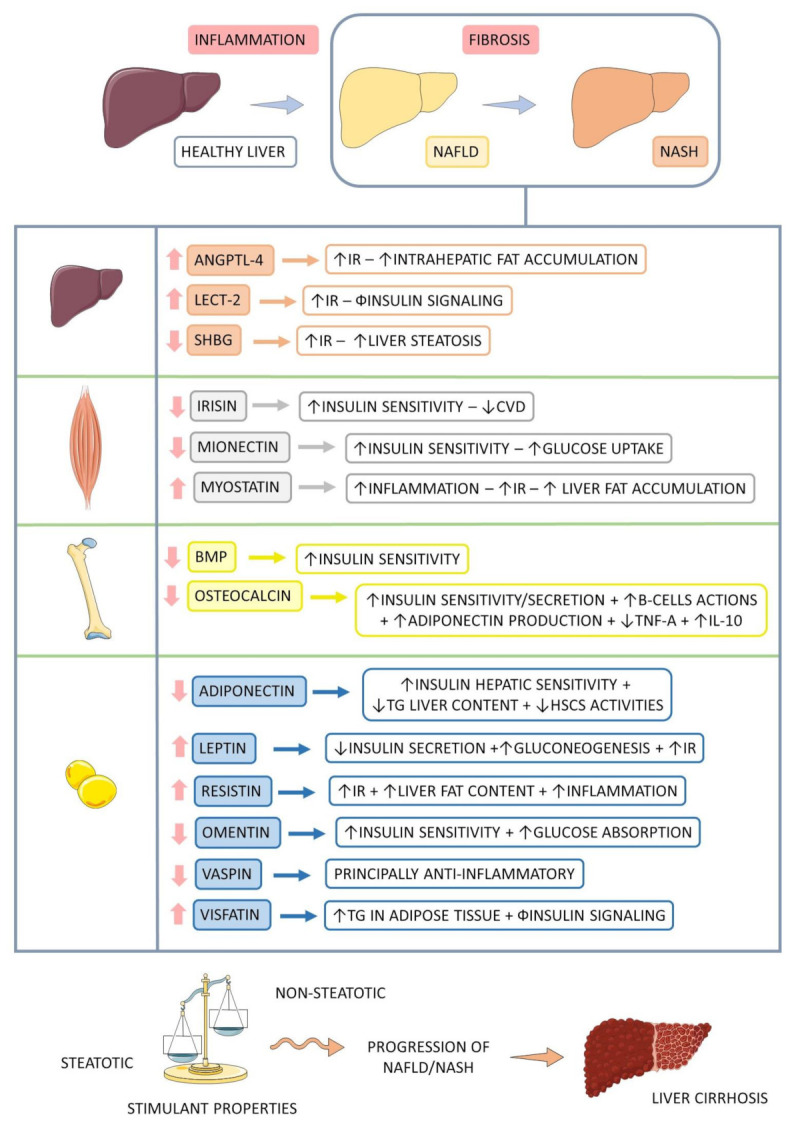
Main adipokines, hepatokines, myokines, and osteokines involved in the pathophysiology of NASH. NAFLD: non-alcoholic fatty liver disease, NASH: non-alcoholic steatohepatitis, ↑: increase, ↓: decrease, Φ: impairment, +: plus, ANGPTL-4: angiopoietin-like 4, IR: insulin resistance, LECT-2: leukocyte cell-derived chemotaxin 2, SHBG: sexual hormone binding globulin, CVD: cardiovascular diseases, BMP: bone morphogenetic protein, β: beta, TNF-α: tumor necrosis factor alfa, IL-10: interleukin 10, TG: triglycerides, HSCs: hepatic stellate cells.

OCN also has a relationship with the nervous system. Its absence makes the hippocampus smaller and less developed, in addition to causing anxiety and memory impairment, while its presence would be effective against age-related decline in cognition and helpful in responding to acute stress, inhibiting the parasympathetic system [84,85,86]. Therefore, OCN stimulates the consumption of glucose and FFAs by promoting the expression of fatty acid transporters, stimulating B-oxidation and translocation of GLUT4 to the plasma membrane. This scenario generates an anti-inflammatory environment, contributing to reducing visceral fat and reducing NASH [75,84,87]. Figure 5 shows the main organokines involved in the pathophysiology of NASH.

### 3.5. Organokines: A Cross-Talk

#### 3.5.1. Apelin

Apelin is produced by fat tissue and muscles and decreased in obesity. Furthermore, it is expressed in the central nervous system (hypothalamus), heart, and stomach. It has an important anti-inflammatory role, helping to control the heart muscle and, in turn, blood pressure, in addition to angiogenesis and cell cycle control and death. This organokine also stimulates age-related muscle regeneration, preventing muscle loss [64,88].

It also regulates glucose metabolism, lipolysis, cardiovascular homeostasis, decreasing lipolysis, and inducing vasodilation, which favors the reduction of blood pressure. In contrast, higher circulating levels indicate apelin resistance and are associated with obesity and IR [60].

#### 3.5.2. Chemerin

Chemerin is considered both adipokine and hepatokine. It is mainly expressed in WAT and is related to the regulation of glucose uptake, lipolysis, and adipocyte differentiation. It is activated by inflammatory and coagulation proteases [64,89] and is considered a chemoattractant for dendritic cells and macrophages. In addition, it regulates bone metabolism through testosterone synthesis and the balance between osteoblasts and osteoclasts [89].

This organokine increases glucose tolerance and hinders insulin signaling, in addition to playing an important role both in bone degeneration and formation, as well as in stimulating inflammation [64,89]. It is increased in NASH since it can represent a link between obesity and inflammation. Its levels are directly associated with elements of MS, such as BMI, hypertension, high triglycerides, and C-reactive protein levels. Chemerin is elevated in obese and DM2 individuals and is inversely related to HDL-c and omentin levels [68,90].

#### 3.5.3. Fetuin-A

Fetuin-A is both hepatokine and adipokine, and its high levels are associated with obesity, IR, DM, and NAFLD, in addition to inhibiting ectopic calcium deposition. Fetuin A also promotes the secretion of pro-inflammatory biomarkers in monocytes and adipocytes and down-regulates the expression of the adiponectin, that is an insulin sensitizing hormone [58,63,77,78].

It is a natural inhibitor of insulin receptors in the liver and skeletal muscle, promoting pro-adipogenic effects, as its high concentration interferes with the insulin signaling cascade and the translocation of GLUT-4 [63,64]. Hepatocytes secrete fetuin-A in the blood, which binds to the insulin receptor in tissues, inhibiting its signaling, and, due to this, induces IR [78]. Furthermore, fetuin-A stimulates the secretion of inflammatory cytokines in monocytes and adipose tissue [77]. Physical activity reduces its levels, with beneficial potential due to the reduction of available FFAs, decreasing visceral adipose tissue mass [64].

#### 3.5.4. Fibroblast Growth Factor 21 (FGF-21)

FGF-21 is released by adipose tissue and liver and is a regulator of glucose and lipid metabolism. Its increased levels in NAFLD correlate with the amount of hepatic triglycerides; therefore, FGF-21 is considered an emerging biomarker of NAFLD. This organokine is a potential therapeutic agent to combat IR, as it adjusts insulin sensitivity, body weight, lipid profile, hepatic IR and may have a role in inflammatory modulation [2,16]. Furthermore, it stimulates the darkening of white adipose tissue [65] due to the increase in the expression of thermogenic genes and adiponectin, fatty acid oxidation in the liver, and insulin sensitivity. FGF-21 increases the oxidation of fatty acids in the liver, reducing glucose production and consequently delaying steatohepatitis development. This hepatokine also stimulates glucose retention in adipocytes due to increased GLUT-1 expression, therefore improving insulin sensitivity. When FGF-21 is released into muscle in response to insulin and increases glucose uptake, it can stimulate bone resorption [57,58,64,77,78].

#### 3.5.5. Follistatin

Follistatin is classified as adipokine and myokine; it is released during exercise, inducing thermogenic genes and neutralizing myostatin [64].

Follistatin is primarily derived from the liver, and its levels increase with an increased glucagon-to-insulin ratio, such as fasting and exercise. Patients with IR due to obesity have higher levels of this organokine. Because it is capable of neutralizing myostatin, follistatin can increase skeletal muscle hypertrophy and induce thermogenic genes in murine adipocytes. Its constant, high levels are related to IR, increased hepatic glucose production, and glucose intolerance. On the other hand, transient increases in follistatin after physical activity may be beneficial because it favors glucose and FFAs uptake by skeletal muscle cells and lipolysis in WAT [75].

#### 3.5.6. IL-6

IL-6, a pro-inflammatory and pro-oncogenic cytokine, is considered adipokine, hepatokine, and myokine. It is a predictive marker of IR and CVD. It suppresses adiponectin levels along with TNF-α and stimulates leptin production [9,64]. It is produced by macrophages and stimulated by NFκβ activation. IL-6 inhibits the expression of insulin receptor substrate and GLUT 4 in adipocytes [64]. While IL-6 derived from adipose tissue stimulates inflammation, glucose intolerance, and bone resorption, IL-6 derived from myocytes has an anti-inflammatory action, as it stimulates the release of IL-10 creating an anti-inflammatory environment, increasing glucose uptake, WAT lipolysis, darkening of adipocytes and improvement of metabolism [57,64,75,89].

#### 3.5.7. Lipocalin 2 (LCN-2)

LCN-2, first recognized as adipokine and later also as osteokine, is expressed by osteoblasts and adipocytes and contributes to satiety, in addition to stimulating energy expenditure. Obese people can have resistance to LCN-2, and its levels increase with aging and decrease with energy expenditure [57,64].

In an animal model with diet-induced NASH, liver damage, and inflammation, it was seen that LCN-2 levels were increased. This change was associated with an increase in intrahepatic neutrophils and the production of pro-inflammatory chemokines, which contributed to the maintenance of the inflammatory process [91]. Thus, LCN-2 is an inflammatory biomarker and is associated with infections, ischemia, kidney damage, intestinal inflammation and metabolic inflammation, and disorders such as DM2 and MS [92]. 

#### 3.5.8. Osteonectin

Osteonectin is recognized as both adipokine and myokine and is also named secreted protein, acidic, and rich in cysteine (SPARC). It modulates the expression of pro-inflammatory cytokines involved in IR adipogenesis, stimulates insulin release and erythropoiesis, and plays an important role in preventing mitotic clonal expansion of preadipocytes, inhibiting the initiation of adipogenesis. Due to inhibition of adipocyte production, it restricts lipid storage, increasing circulating levels and leading to systemic hyperlipidemia and secondary fat deposition in the liver and skeletal muscle [64]. 

Mazzolini et al. [93] associated low levels of this organokine as a protective factor against NASH and reduced liver damage in morbidly obese patients. In turn, its absence has been related to alterations in the hepatic lipid metabolism and the risk of developing hepatocellular carcinoma, which is closely related to NAFLD [94].

The reduction of FGF-21 levels in adult subjects with severe liver steatosis are due to the injury or death resulted from lipotoxicity and hepatic inflammation. This reduction may also be related to the disease progression to NASH. The augment in FGF-21 production could be a potential protection factor against carbohydrates and lipids metabolism disorders since this hepatokine directly regulates lipid metabolism and hepatic accumulation in an insulin-independent manner. Moreover, it interferes with several elements of the multi-hits theory of NAFLD such as mitochondrial dysfunction, oxidative stress, low-grade chronic inflammation, and endoplasmic reticulum stress [95].

#### 3.5.9. Selenoprotein-P (SeP)

Selenoprotein-P is produced by both the liver and adipose tissue and contributes to IR in DM2 [75,77]. It acts as an intracellular antioxidant in phagocytes, modulating the inflammatory response through the differentiation of macrophages from M1 to M2, reducing oxidative damage and, therefore, preventing damage to hepatic endothelial cells.

The literature is quite conflicting about their levels in the face of the inflammatory response, including in metabolic diseases and the presence of NAFLD and NASH [63,64,74,75].

#### 3.5.10. TNF-α 

TNF- *α* is a cytokine contributing steatosis, oxidative stress, and inflammatory mediator cytokine, released by numerous tissues and being an inducer of hepatocyte death. The high increase in TNF-α is associated with obesity and IR, through the up-regulation of SOCS3 (suppressor of cytokine signaling 3) [96]. Hepatocytes possess TNF superfamily death receptors, which are activated by TNF-*α* during the progression from NAFLD to NASH. When it binds to TNF receptor 1 (TNFR1), it induces the activation of caspases 8 and 3, leading to apoptosis of liver cells, which is an important process for the degradation of damaged cellular components. In cases of stress, such as in inflammatory liver diseases, apoptosis occurs to preserve cell function, but its occurrence in excess can lead to irreversible cell atrophy and its collapse, thus contributing to the progression of NASH [97]. TNF-α increases lipolysis, thus increasing serum free fatty acids and thus favoring the development of insulin resistance. Furthermore, by stimulating and activating vascular adhesion molecules, it favors atherogenesis. Another mechanism involved in TNF-α induction of insulin resistance in peripheral tissues is the activation of nuclear factor-κB (NF-κB) and stimulation of the transcription of cytokines and adhesion molecules. Additionally, it acts as a chemoattractant for monocytes and neutrophils. It increases the cytotoxicity of monocytes and macrophages, being at the same time one of the mediators of cytotoxicity. Its numerous functions are mediated, among others, by its ability to induce the synthesis of other cytokines functionally associated with TNF-α, extracellular matrix proteins, monocyte modulation and fibroblast chemotaxis, as well as impact on the expression of vascular adhesion molecules. Therefore, due to its numerous pro-inflammatory effects, it contributes to the evolution of NASH [68].

#### 3.5.11. TGF-β

TGF-β is a pleiotropic cytokine involved in survival, proliferation, differentiation, angiogenesis and wound healing response. There are studies suggesting that hepatocytes with excess of lipids increase the production of ROS mediated by TGF-β, resulting in increased hepatocyte death. Thus, TGF-β signaling in hepatocytes contributes to hepatocyte death, lipid accumulation and ROS production that promote the development of NASH. In the liver, TGF-β signaling participates in the fibrogenic response through the activation of hepatic stellate cells (HSC). Therefore, TGF-β signaling in HSC plays a role in the progression of fibrosis in advanced NAFLD [98]. 

**Figure 6:**
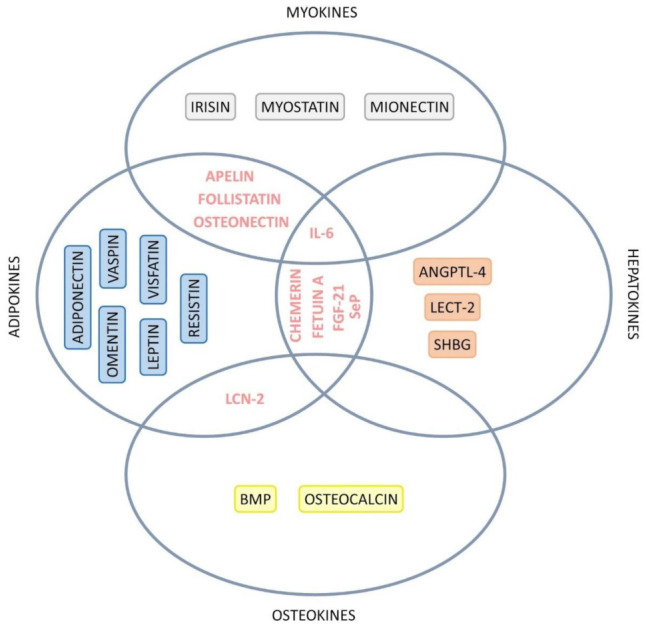
Venn diagram showing the organokines with simultaneous classification involved in the pathophysiology of NASH. IL-6: interleukin 6, FGF-21: fibroblast growth factor 21, SeP: selenoprotein-P, ANGPTL-4: angiopoietin-like 4, LECT-2: leukocyte cell-derived chemotaxin 2, SHBG: sexual hormone binding globulin, LCN-2: lipocalin 2, BMP: bone morphogenetic protein.

Table 1 summarizes the organokines that have been linked to NASH development. Additionally, Figure 6 summarizes the organokines with simultaneous classification involved in the pathophysiology of NASH.

Table 2 shows the main characteristics of organokines with simultaneous classification involved in the pathophysiology of NASH.

## 4. Paths to Unravel

Several exciting questions can arise since these organokines affect each other and communicate through various endocrine, paracrine and autocrine pathways. It is known that several environmental factors (such as diet, exercise, stress, sleep quality, and microbiota) and genetic factors are related to the expression of organokines, but how much and what lifestyle changes can modulate the expression of these mediators?

How do patients with NASH, with some degree of therapeutic intervention (diet, physical activity, pharmacological treatment) differ qualitatively and quantitatively in producing these organokines (hepatokines) compared to sedentary and untreated patients?

### 4.1. Genetics

How can individual genetic variations interfere in the production (expression, synthesis) of these organokines and, therefore, the susceptibility to developing metabolic diseases of the liver and other organs?

### 4.2. Diet and Microbiota

Some dietary compounds are metabolized by the gut microbiota and result in metabolites capable of modulating the host metabolism. On the other hand, diet can modify the microbiota composition and consequently exert beneficial or harmful effects on the host. These interferences can be localized in the gut, but others have systemic effects, leading, for example, to IR and can be related to the MetS risk factors [99]. Furthermore, diet regulates some organokines, such as FGF-21 [100]. 

Some authors have also shown the link between the microbiome, NAFLD and NASH pathogenesis and the interaction between the gut and liver, named the gut–liver axis, play a critical role in NASH development and evolution [101,102]. Microbiome-derived components can affect the liver via biomarkers that lead to interconnected effects such as gene expression, pro-inflammatory signaling, and modifications in metabolism and toxicity, resulting in liver inflammation and fibrosis [101]. 

We can think of several associations: diet interferes with the microbiota, which interferes with the production of metabolites related to MetS. Diet high in sugars and fats alone is associated with MetS (a condition directly associated with NAFLD/NASH). In a liver inflammation scenario, the secretion of organokines (especially hepatokines) may be altered, thus altering the crosstalk between other organokines and possibly amplifying the process of a vicious cycle. Therefore, is there a possibility that the intestinal microbiota modulates the synthesis of organokines/hepatokines? How does the microbiota interfere with the expression of hepatokines in patients with liver disease? If the gut liver axis is involved with the pathogenesis and progression of NASH, most likely the composition or changes in the gut microbiota influence the establishment of NASH and indirectly the secretion of hepatokines. As hepatokines participate in crosstalk, then indirectly, the metabolism of other organs/tissues such as the pancreas, adipose tissue, and muscle may also be affected.

### 4.3. Exercise

Some authors have shown that exercise and maintained muscle mass can modify the circulating amounts of several organokines. Exercise can prevent liver steatosis and fibrosis in patients with NAFLD, independent of weight loss. The benefits of exercise seem to be associated with modifying inter-organs crosstalk, resulting in organokine balance and decreased inflammation and oxidative stress [103].

### 4.4. Sleep, Melatonin and Organokines

It is known that there is a relationship between physical activity and melatonin release, which in turn influences both the composition of skeletal muscle (type of fibers) and the metabolism of this tissue. Since skeletal muscle secretes myokines and crosstalks with other organs, we believe that there is a possible causal relationship between melatonin production, organokine secretion and the degree of inflammation. This set would be related to the development of NAFLD/NASH. 

Sleep restriction and poor sleep quality are profoundly implicated in the pathogenesis of metabolic dysfunctions, including NAFLD [104]. In the presence of NASH or NAFLD, it is plausible to admit that it will have an alteration in the release of hepatokines. Consequently, it may alter the release of other organokines and crosstalk.

Some authors have explored the relationship between melatonin and skeletal muscle metabolism [105]. As we know, muscle secretes myokines with different metabolic effects. The question is: is there a link between melatonin and myokine secretion? If it exists, can endogenous production or therapeutic use of melatonin interfere with organokine production? Could this have any therapeutic benefit? How can sleep quality interfere qualitatively and quantitatively on the output of organokines/hepatokines?

### 4.5. Other Metabolic Conditions

It is plausible that other conditions such as liver iron overload (hyperferritinemia/dysmetabolic iron overload syndrome) can alter the synthesis of these hepatokines and consequently crosstalk and thus play a role in the pathogenesis or progression of NASH (circuit and feedback)?

### 4.6. Intervention, Management, and Therapy 

As discussed above, for the development of NASH, the two hits hypothesis is accepted. Most organokines mentioned in this review participate in the first or second hit and therefore influence the onset of liver disease. Besides that, as organokines may be involved in the origin of NAFLD and its progression to NASH, it is possible to use the dosage of these biomarkers as a marker for liver disease and its progression.

For future perspectives, we can think if there is a possibility of interfering pharmacologically in the synthesis of organokines. Could this represent a therapeutic strategy to control diabetes, obesity, hypertension, MetS, CVD, and NASH? 

## 5. General Comments

Insulin resistance, inflammation and mitochondrial dysfunction, fructose and intestinal microbiota were factors identified as participating in the genesis and progression of NASH. In this context, modifications in the pattern of organokine (adipokines, myokines, hepatokines, and osteokines) secretion are observed, which directly or via crosstalk contribute to aggravating the condition.

Chronologically, the focus has been on studying the endocrine role of adipose tissue and muscle. Recent findings show new soluble factors secreted by the liver, bones, and pancreas. In the future, we believe that this organ list will increase. Thus, this extensive and complex network of information established by crosstalk will be amplified, thus improving our understanding of how organokines interact and how this interaction can open pathways for intervention in diseases like NASH. 

Obesity, MetS, DM2, CVD, and NAFLD are interconnected conditions, and without a doubt, the different organokines act in a joint and coordinated way in the origin and progression of these diseases. Especially in NASH, the participation of these soluble factors is clear, both for their protective effects and their potentiating effects (development and amplification of the inflammatory process). The development of new and promising therapies, potentially helpful in stabilizing liver disease, will necessarily involve understanding the synergistic, antagonistic, and pleiotropic effects of these organokines on metabolism and inflammatory processes.

## Figures and Tables

**Table 1:** Main characteristics of adipokines, hepatokines, myokines, and osteokines involved in the pathophysiology of non-alcoholic steatohepatitis.

Release	Organokine	Expression	General Function	Role in NASH	Reference
ADIPOKINE	Adiponectin	↓	Anti-inflammatory; Increases fatty acid oxidation; Increases glucose uptake in skeletal muscle; Inhibition of NFκβ and TNF-α by secreting IL-10; IR-related plasma reduction and glucose intolerance.	Hepatic insulin-sensitizing effect; Prevention of TG accumulation in the liver; Maintenance of HSCs in their quiescent state.	[57,61,63,64,65,66]
	Leptin	↑	Weight loss increased energy expenditure and FFA oxidation; Increases glucose and FFA absorption in skeletal muscle; Stimulates production of IL-6 and TNF-α; Reduction of appetite and TAG synthesis; Neutralization of lipogenic action of insulin; Pro-inflammatory action; NO release and increases endothelin.	Reduction of insulin secretion; Failure in the antisteatotic effect; Increase of gluconeogenesis and IR.	[57,63,64,67]
	Omentin	↓	Anti-atherosclerotic effects.	Improvement of insulin sensitivity and glucose absorption.	[64,68,69]
	Resistin	↑	Pro-inflammatory effects.	Induction of inflammation, IR, angiogenesis, and smooth muscle cell proliferation; Reduction of mitochondria content and increases fat accumulation.	[9,58,64,65,70]
	Vaspin	↓	Anti-inflammatory effects.	Inhibition of kallikrein 7; Reduction of insulin sensitivity; Reduce the synthesis of pro-inflammatory cytokines.	[60,64]
	Visfatin	↑	Dysfunction of pancreatic β cells, Activation of leukocytes; Production of pro-inflammatory cytokines in adipose tissue.	Stimulation of the production and storage of triacylglycerols in adipose tissue; Impair insulin signaling.	[64,65,68]
HEPATOKI-NE	ANGPTL-4	↑	Induction of IR in the liver, skeletal muscle, and adipose tissue.	Accumulation of liver lipids.	[16,75,79]
	LECT-2	↑	Promotion of IR in skeletal muscle by activating JNK.	Impairment of insulin signaling.	[16,64]
	SHBG	↓	Association with systemic IR.	Suppression of lipogenesis in the liver, exacerbating steatosis.	[16,64]
MYOKINE	Irisin	↓	Energy homeostasis and interactions between skeletal muscle and other tissues; Darkening of the WAT; Suppression of adipogenesis and cholesterol synthesis; Increase in lipid oxidation.	Increase of insulin sensitivity and improvement of Metabolic Syndrome and CVD.	[57,63,64,73]
	Mionectin	↓	Increase of lipid uptake by adipose tissue and liver.	Insulin sensitivity.	[64]
	Myostatin	↑	Pro-inflammatory effects; Reduction of muscle mass, increasing metabolic disorders.	Increase of IR and liver fat deposition.	[64,76]
OSTEOKINE	BMP	↓	Darkening of the WAT and increase of thermogenesis; Stimulation of insulin secretion.	Improvement of insulin sensitivity.	[57,83]
	Osteocalcin	↓	Stimulation of glucose uptake and increase in insulin sensitivity; It increases the glucose and FFA uptake by muscles; Muscle hypertrophy and strength.	Increase of sensitivity and insulin secretion; Survival and functioning of pancreatic β cells; Stimulates the production of adiponectin and IL-10; Reduces TNF-α levels in adipocytes	[57,75,84,87]

↑: increase, ↓: decrease, TG: triglycerides, HSCs: hepatic stellate cells, NF-kB: nuclear factor kappa b, TNF-α: factor tumor necrosis alfa, IL-10: interleukin 10, IR: insulin resistance, FFA: free fatty acids, IL-6: interleukin 6, NO: nitric oxide, TAG: triacylglycerol, β: beta, WAT: white adipose tissue, CVD: cardiovascular diseases, ANGPTL-4: angiopoietin-like 4, LECT-2: leukocyte cell-derived chemotaxin 2, SHBG: sexual hormone binding globulin and BMP: bone morphogenetic protein.

**Table 2:** -Main characteristics of organokines with simultaneous classification involved in the pathophysiology of non-alcoholic steatohepatitis (NASH).

Organokine	Classification	Expression	General Actions	Role in NASH	Reference
Apelin	Adipokine and myokine	↓	Control of cardiac muscle; Control of the cycle and cell death; Muscle regeneration.	Anti-inflammatory.	[64,88]
Chemerin	Adipokine and hepatokine	↑	Increase in glucose tolerance and hinders insulin signaling.	Impairment of glucose homeostasis.	[64,90]
Fetuin A	Adipokine and hepatokine	↑	Inhibition of insulin receptors in the liver and skeletal muscle; Pro-adipogenic; Suppression of adiponectin; IR in the liver via ER stress and JNK activation.	Association with NAFLD severity; IR and accelerates atherogenesis; Association with fatty liver.	[58,63,64,77,78]
FGF-21	Adipokine and hepatokine	↑	Lipolysis; Stimulates adiponectin, insulin sensitivity, fatty acid oxidation in the liver, and glucose retention in adipocytes; Decrease of glucose production; Insulin secretion.	Modulates oxidative and ER stress, mitochondrial dysfunction, and low-grade inflammation to improve NAFLD; Increase of hepatic fat oxidation; Decrease of adipose tissue lipolysis.	[20,57,58,64,77,78]
Follistatin like-1	Adipokine and myokine	↑	Stimulation of bone regeneration.	Stimulation of IL-1β production; Stimulation of fibrosis development.	[57]
IL-6	Adipokine, hepatokine, and myokine		From adipocytes: pro-inflammatory effects; From myocytes: anti-inflammatory actions.	Adipocytes: RI; Myocytes: insulin sensitization.	[57,64]
LCN-2	Adipokyne and osteokyne	↓	Satiety and stimulation of energy expenditure.	Production of pro-inflammatory chemokines.	[57,64]
Osteonectin	Adipokine and myokine	↑	Modulates expression of pro-inflammatory cytokines.	IR; Adipogenesis.	[64]
SeP	Adipokine and hepatokine	↑	Transportation of selenium from the liver to the rest of the body; Contribution to IR, impairing insulin signaling in hepatocytes.	IR	[16,63,64,77]

↑: increase, ↓: decrease, NAFLD: non-alcoholic fatty liver disease, NASH: non-alcoholic steatohepatitis, FGF-21: fibroblast growth factor 21, IL-6: interleukin-6, LCN-2: lipocalin-2, SeP: selenoprotein-P, IR: insulin resistance, ER: endoplasmic reticulum stress, JUNK: c-Jun N-terminal cinase and IL-1β: interleukin 1 beta.
